# Associations between common ECG abnormalities and out-of-hospital cardiac arrest

**DOI:** 10.1136/openhrt-2018-000905

**Published:** 2019-05-21

**Authors:** Marc Meller Søndergaard, Jonas Bille Nielsen, Rikke Nørmark Mortensen, Gunnar Gislason, Lars Køber, Freddy Lippert, Claus Graff, Stig Haunsø, Jesper Hastrup Svendsen, Kristian Hay Kragholm, Adrian Holger Pietersen, Bent Struer Lind, Søren Pihlkjær Hjortshøj, Anders Gaarsdal Holst, Johannes Jan Struijk, Christian Torp-Pedersen, Steen Møller Hansen

**Affiliations:** 1 Department of Clinical Epidemiology, Aalborg Universitetshospital, Aalborg, Denmark; 2 Division of Cardiovascular Medicine, Department of Internal Medicine, University of Michigan, Ann Arbor, Michigan, USA; 3 Department of Human Genetics, University of Michigan, Ann Arbor, Michigan, USA; 4 Department of Cardiology, Herlev Hospital, Herlev, Denmark; 5 National Institute of Public Health, University of Southern Denmark, Odense, Syddanmark, Denmark; 6 Department of Cardiology, Rigshospitalet, Kobenhavn, Denmark; 7 Emergency Medical Services Copenhagen, University of Copenhagen, Kobenhavns, Denmark; 8 Department of Health, Science and Technology, Aalborg Universitet Det Sundhedsvidenskabelige Fakultet, Aalborg, Denmark; 9 Laboratory of Molecular Cardiology, Department of Cardiology, The Heart Centre, Rigshospitalet, Kobenhavn, Denmark; 10 Department of Cardiology, The Heart Centre, Rigshospitalet, Kobenhavn, Denmark; 11 Laboratory for Molecular Cardiology, The Heart Centre, Rigshospitalet, Kobenhavn, Denmark; 12 Department of Cardiology, Regionshospital Nordjylland, Hjorring, Nordjylland, Denmark; 13 Department of Cardiology, Nephrology and Endocrinology, Nordsjaellands Hospital, Hillerod, Denmark; 14 Department of Clinical Biochemistry, Hvidovre Hospital, Hvidovre, Denmark; 15 Department of Cardiology, Aalborg Universitetshospital, Aalborg, Denmark

**Keywords:** ECG, out-of-hospital cardiac arrest, cardiac disease, risk

## Abstract

**Background:**

Out-of-hospital cardiac arrest (OHCA) is often the first manifestation of unrecognised cardiac disease. ECG abnormalities encountered in primary care settings may be warning signs of OHCA.

**Objective:**

We examined the association between common ECG abnormalities and OHCA in a primary care setting.

**Methods:**

We cross-linked individuals who had an ECG recording between 2001 and 2011 in a primary care setting with the Danish Cardiac Arrest Registry and identified OHCAs of presumed cardiac cause.

**Results:**

A total of 326 227 individuals were included and 2667 (0,8%) suffered an OHCA. In Cox regression analyses, adjusted for age and sex, the following ECG findings were strongly associated with OHCA: ST-depression without concomitant atrial fibrillation (HR 2.79; 95% CI 2.45 to 3.18), left bundle branch block (LBBB; HR 3.44; 95% CI 2.85 to 4.14) and non-specific intraventricular block (NSIB; HR 3.15; 95% CI 2.58 to 3.83). Also associated with OHCA were atrial fibrillation (HR 1.89; 95% CI 1.63 to 2.18), Q-wave (HR 1.75; 95% CI 1.57 to 1.95), Cornell and Sokolow-Lyon criteria for left ventricular hypertrophy (HR 1.56; 95% CI 1.33 to 1.82 and HR 1.27; 95% CI 1.12 to 1.45, respectively), ST-elevation (HR 1.40; 95% CI 1.09 to 1.54) and right bundle branch block (HR 1.29; 95% CI 1.09 to 1.54). The association between ST-depression and OHCA diminished with concomitant atrial fibrillation (HR 1.79; 95% CI 1.42 to 2.24, p < 0.01 for interaction). Among patients suffering from OHCA, without a known cardiac disease at the time of the cardiac arrest, 14.2 % had LBBB, NSIB or ST-depression.

**Conclusions:**

Several common ECG findings obtained from a primary care setting are associated with OHCA.

**Key questions**

**What is already known about this subject?**

- About 50% of out-of-hospital cardiac arrests (OHCAs) occur as the first manifestation of previously unrecognised heart disease. This challenges preventive steps for identifying patients at risk. Various ECG abnormalities have been associated with an increased risk of sudden cardiac death but routine ECG screenings of populations are not recommended. In primary care settings, ECG examinations are very common. Consequently, when an ECG abnormality is encountered, knowledge about the associated risk of OHCA is important as patients could be referred for secondary care evaluation and ultimately treatment for an underlying heart disease.

**What does this study add?**

- Using a large contemporary primary care population referred for ECG recording, we examined the risk of OHCA of presumed cardiac cause based on commonly encountered ECG abnormalities. We found that several of such commonly encountered ECG abnormalities conferred a significantly increased absolute risk of OHCA. Especially left bundle branch block (LBBB), non-specific intraventricular block (NSIB) and ST-depression without concomitant atrial fibrillation were important risk markers.

**How might this impact on clinical practice?**

- When ECG findings are encountered in primary care settings such as LBBB, ST-depression without concomitant atrial fibrillation and NSIB, preventive measures such as referring the patient to a secondary care evaluation should be considered.

## Introduction

Cardiac arrest and sudden cardiac death are commonly observed among persons with established cardiovascular disease.^1^ It has been reported, however, that 40%–60% of all sudden cardiac deaths occur as the first manifestation of previously unrecognised heart disease. This challenges potential preventive steps for patients at high risk of suffering an out-of-hospital cardiac arrest (OHCA).^2 3^ The ECG is an inexpensive and widely available examination for detecting cardiac diseases, and several studies have shown that ECG findings such as left ventricular hypertrophy (LVH), left bundle branch block (LBBB), pathological Q-waves and ST-segment deviations are associated with increased cardiac morbidity and mortality.^4–10^ Routine ECG screening of asymptomatic persons is not recommended.^3 11^ Nevertheless, in a primary care setting, ECG examinations are commonly performed on a routine basis on a large variety of patients. Consequently, knowledge about risks of cardiac arrest related to commonly encountered ECG findings is important as this could prove a tool for identifying otherwise unidentified high-risk patients.

Using a large contemporary primary care population referred for ECG recording, we examined the risk of OHCA of presumed cardiac cause based on commonly encountered ECG abnormalities.

## Methods

We studied individuals from the Copenhagen ECG study, comprising a large cohort of primary care patients referred to a central core facility (Copenhagen General Practitioners’ Laboratory (CGPL)) for digital ECG recordings, from 1 June 2001 to 26 September 2011. Further details on the population have been described previously.^12^

### Out-of-hospital cardiac arrests

OHCAs were identified using the Danish Cardiac Arrest Registry from 1 June 2001 until 31 December 2012.^13^ The Cardiac Arrest Registry is a nation-wide registry and includes clinical conditions of cardiac arrest outside of hospital that resulted in a resuscitative effort either by bystanders (an individual who witnessed the collapse or found the person unresponsive) or by emergency medical service personnel. Only first-time OHCAs of presumed cardiac cause were considered in this study. Presumed cardiac cause of the arrest was defined using death certificates and/or discharge diagnoses consistent with cardiac disease, unknown disease or unexpected collapse. Death certificates and discharge diagnoses with other causes defined a non-cardiac cause of arrest, including trauma, drug overdose, suicide, and violent attack.^13^ Patients suffering a cardiac arrest of presumed non-cardiac cause were included in the cohort and followed as the rest of the population.

### ECG analysis and interpretation

All ECGs were digitally recorded and stored in the MUSE Cardiology Information System (GE Healthcare, Wauwatosa, WI, USA). The ECGs were processed by the Marquette 12SL algorithm, V.21.

We used the 12SL algorithm to identify atrial fibrillation, atrial flutter, LBBB, right bundle branch block (RBBB) and 12SL measurements to code the following composite ECG criteria: Q-waves, ST-deviations, Sokolow-Lyon and Cornell voltage criteria for LVH and non-specific intraventricular block (NSIB).^14^ ECGs with NSIB were identified as ECGs with QRS duration >120 ms without fulfilling criteria for RBBB or LBBB. Significant ST-T deviations and pathologic Q-waves (as a sign of prior myocardial infarction) were defined according to the Third Universal Myocardial Infarction Definition.^15^ The definition of ST-elevation was slightly modified as the measurement of the ST segment was performed at QRS offset plus 1/16 of the average RR interval known as the STM point measure in the 12SL algorithm (equivalent to about 80 ms after QRS offset in most cases). This measurement point was selected instead of the J-point because a notched or slurred appearance of the terminal QRS complex (also described as early repolarisation) can make it difficult to define the J-point.^12^ LBBB and NSIB are known to affect the repolarisation of the heart causing ST-deviations.^15–17^ Consequently, when ST-T deviation was concomitantly present with LBBB or NSIB, we disregarded the finding. In patients with RBBB, ST-T deviations in V1–V3 are common.^15^ When ST-T deviations in V1–V3 were present together with RBBB, the ST-T deviations were disregarded.

The Sokolow-Lyon and Cornell sex-specific voltage criteria were used to identify ECG LVH.^18 19^ The criteria for hypertrophy have low predictive value when applied on an ECG with identified LBBB, RBBB and NSIB.^20^ ST-T deviations together with ECGs with hypertrophy have been associated with larger left ventricular mass and risk of cardiovascular disease.^20^ Consequently, if LBBB, RBBB or NSIB were identified, ECGs were not assigned the Sokolow-Lyon and Cornell hypertrophy criteria. ST-T deviations together with the hypertrophy criteria were acknowledged and included in the analysis. In accordance with the Third Universal Myocardial Infarction Definition, Q-waves, as a sign of prior myocardial infarction, were only defined when hypertrophy criteria or LBBB were absent.^15^

### Patient characteristics, comorbidities and medication

In Denmark, all citizens have a unique civil registration number enabling linkage of information between various national registries on an individual level. Using the civil registration number for our study participants, we obtained data regarding age, sex and vital status from The Danish National Population Registry. We used discharge and outpatient diagnoses from the Danish National Patient Registry to classify patient comorbidity prior to the start of follow-up on an individual level. Causes of death were collected from the Danish Registry of Causes of Death. All discharge diagnoses and causes of death were classified according to the WHO International Classification of Diseases, 10th Edition. Medicine use was collected from the Danish National Prescription Registry, which contains all dispensed prescriptions from Danish pharmacies since 1995 and is classified according to the Anatomical Therapeutic Chemical System.

We identified patient comorbidity up until 5 years before the baseline ECG recording and redemption of prescriptions up until 180 days before the ECG recording. Cardiac diseases were defined from cardiac disease diagnosis, including ischaemic heart disease, previous myocardial infarction, cardiomyopathy, heart failure, valvular heart disease, atrial fibrillation, atrial flutter, congenital heart disease, other cardiac arrhythmias and other cardiac diseases (online supplementary material, table S1). As non-cardiac comorbidities, we included cerebrovascular disease, peripheral vascular disease, malignant disease, renal disease, liver disease and chronic obstructive pulmonary disease (online supplementary material, table S1). The data quality in the Danish registries of the diagnoses in this study has previously been shown to be high.^21^ Medication included the following: QTc prolonging medication, glucose lowering medicine, beta-blockers, diuretics, ACE inhibitors/angiotensin receptor blockers and calcium channel blockers (online supplementary material, table S2-S3).

10.1136/openhrt-2018-000905.supp1 Supplementary data

### Study population

We excluded ECGs identified by the 12SL algorithm as poor quality ECGs. ECGs with pacemaker rhythms, as identified by the 12SL algorithm or by an ECG technician or consultant in cardiology at CGPL, were also excluded.^12^ In addition, we excluded ECGs with second or third degree atrioventricular block, ECGs not qualified for sufficient interpretation, ECGs where the patient had a pacemaker or implantable cardioverter-defibrillator implant prior to the ECG acquisition date, patients younger than 16 years, ECGs from patients with missing information regarding sex or cause of arrest and ECGs other than the latest acquired ECG before OHCA or censoring were also excluded. Information on the accuracy of the 12SL algorithm can be found in the online supplementary material.

### Statistics

Dichotomous variables were reported as frequencies with corresponding percentages and continuous variables were reported as medians and first to third quartiles (Q1–Q3). Individuals were followed from the day of the first ECG recording at CGPL (baseline ECG) and until an event of OHCA, death from other cause, emigration or 31 December 2012, whichever came first. Cause-specific Cox regression, with time since ECG recording as time scale, was used to estimate the association of the different baseline ECG findings and OHCA. Results are presented as cause-specific HRs with corresponding 95% CI. The assumption of proportional hazards was evaluated using Schoenfeld residuals and were not violated.

First, a multivariable cause-specific Cox regression analysis was constructed with all ECG criteria in the same model, adjusted for age and sex. We tested for interactions between the various ECG criteria. Only interaction terms with p<0.01 were considered for inclusion in the multivariable model. Second, we performed a multivariable analysis with additional inclusion of comorbidities and medication use, as listed in table 1 and supplementary material, tables S1-S3, except for congenital heart disease due to few events. Third, we estimated person-specific absolute 10-year risks of OHCA for women and men at age 50, 60 or 70 years old, with or without known cardiac disease by combining cause-specific Cox regression models for OHCA and death from other causes.^22^ For these analyses, 95% bootstrap confidence intervals were estimated.

**Table 1:** Characteristics of the study population

Variable	Censored	Death from other cause	Out-of-hospital cardiac arrest	Total
Count, no.	270 825	52 735	2667	326 227
Median age in years (Q1–Q3)	53.1 [40.2, 64.6)	78.8 [69.2, 85.4)	72.5 [62.3, 80.7)	57.1 [43.0, 70.2)
Male sex, no. (%)	122 412 (45.2)	23 229 (44.0)	1650 (61.9)	147 291 (45.1)
Atrial fibrillation	5073 (1.9)	6487 (12.3)	337 (12.6)	11 897 (3.6)
Atrial flutter	502 (0.2)	478 (0.9)	23 (0.9)	1003 (0.3)
Cornell LVH	7570 (2.8)	4916 (9.3)	235 (8.8)	12 721 (3.9)
Sokolow-Lyon LVH	16 801 (6.2)	5486 (10.4)	339 (12.7)	22 626 (6.9)
Left bundle branch block	2037 (0.8)	1741 (3.3)	124 (4.6)	3902 (1.2)
Non-specific intraventricular block	2526 (0.9)	889 (1.7)	109 (4.1)	3524 (1.1)
Right bundle branch block	4346 (1.6)	3213 (6.1)	145 (5.4)	7704 (2.4)
Q-wave	14 093 (5.2)	7106 (13.5)	431 (16.2)	21 630 (6.6)
ST-depression without AF	4585 (1.7)	4635 (8.8)	309 (11.6)	9529 (2.9)
ST-depression with AF	961 (0.4)	2228 (4.2)	117 (4.4)	3306 (1.0)
ST-elevation	2867 (1.1)	889 (1.7)	66 (2.5)	3822 (1.2)
**Cardiac disease**				
Cardiomyopathy	656 (0.2)	491 (0.9)	46 (1.7)	1193 (0.4)
Heart failure	2746 (1.0)	4830 (9.2)	339 (12.7)	7915 (2.4)
Ischaemic heart disease (MI not included)	9884 (3.6)	6167 (11.7)	408 (15.3)	16 459 (5.0)
Previous MI	3285 (1.2)	2134 (4.0)	168 (6.3)	5587 (1.7)
AF/atrial flutter	5339 (2.0)	5872 (11.1)	305 (11.4)	11 516 (3.5)
Valvular heart disease	1034 (0.4)	1189 (2.3)	84 (3.1)	2307 (0.7)
Congenital heart disease	220 (0.1)	29 (0.1)	4 (0.1)	253 (0.1)
Other cardiac arrythmia	3465 (1.3)	1676 (3.2)	83 (3.1)	5224 (1.6)
Other cardiac disease	1148 (0.4)	538 (1.0)	33 (1.2)	1719 (0.5)
**Other diseases**				
Cerebrovascular disease	5967 (2.2)	6005 (11.4)	251 (9.4)	12 223 (3.7)
Peripheral vascular disease	2615 (1.0)	2868 (5.4)	154 (5.8)	5637 (1.7)
COPD	5680 (2.1)	5405 (10.2)	271 (10.2)	11 356 (3.5)
Malignant disease	8101 (3.0)	6206 (11.8)	183 (6.9)	14 490 (4.4)
Renal disease	862 (0.3)	937 (1.8)	45 (1.7)	1844 (0.6)
Liver disease	2223 (0.8)	1000 (1.9)	48 (1.8)	3271 (1.0)
**Medication**				
QTc prolonging medication	62 801 (23.2)	19 338 (36.7)	807 (30.3)	82 946 (25.4)
Glucose lowering medication	15 344 (5.7)	5684 (10.8)	369 (13.8)	21 397 (6.6)
Beta-blockers	26 436 (9.8)	10 183 (19.3)	629 (23.6)	37 248 (11.4)
Diuretics	41 951 (15.5)	25 643 (48.6)	1300 (48.7)	68 894 (21.1)
ACEi/ARB	48 444 (17.9)	14 989 (28.4)	960 (36.0)	64 393 (19.7)
Calcium inhibitors	27 745 (10.2)	10 821 (20.5)	639 (24.0)	39 205 (12.0)

All results are reported as the number of patients (%) unless otherwise specified.

ACEi/ARB, ACE inhibitor/angiotensin II receptor blockers; AF, atrial fibrillation; COPD, chronic obstructive pulmonary disease; LVH, left ventricular hypertrophy; MI, myocardial infarction; Q1–Q3, 1st+3rd quartiles.

**Table 2:** ECG abnormalities and patient characteristics for patients suffering from out-of-hospital cardiac arrest according to whether the patients had cardiac disease when the cardiac arrest occurred

Variable	Out-of-hospital cardiac patients with cardiac disease*	Out-of-hospital cardiac patients without cardiac disease*	Total
Count, no.	1315	1352	2667
Median age in years (Q1–Q3)	75.3 [65.5, 82.3)	69.5 [59.6, 78.4)	72.5 [62.3, 80.7)
Male sex, no.	834 (63.4)	816 (60.4)	1650 (61.9)
Median follow-up (Q1–Q3)	1.9 [0.7, 4.0)	1.7 [0.6, 3.7)	1.8 [0.6, 3.8)
Atrial fibrillation	287 (21.8)	50 (3.7)	337 (12.6)
Atrial flutter	20 (1.5)	3 (0.2)	23 (0.9)
Cornell LVH	145 (11.0)	90 (6.7)	235 (8.8)
Sokolow-Lyon LVH	185 (14.1)	154 (11.4)	339 (12.7)
Left bundle branch block	77 (5.9)	47 (3.5)	124 (4.6)
Non-specific intraventricular block	71 (5.4)	38 (2.8)	109 (4.1)
Right bundle branch block	89 (6.8)	56 (4.1)	145 (5.4)
Q-wave	259 (19.7)	172 (12.7)	431 (16.2)
ST-depression without atrial fibrillation	202 (15.4)	107 (7.9)	309 (11.6)
ST-depression with atrial fibrillation	99 (7.5)	18 (1.3)	117 (4.4)
ST-elevation	46 (3.5)	20 (1.5)	66 (2.5)

All results are reported as the number of patients (%) unless otherwise specified.

Q1–Q3 1st+3rdquartiles.

*Cardiac disease at the time of the cardiac arrest: heart failure, ischaemic heart disease, prior myocardial infarction, cardiomyopathy, atrial fibrillation, atrial flutter, congenital heart disease, valvular heart disease, other cardiac arrhythmia, other cardiac disease.

LVH, left ventricular hypertrophy.

Data management and analysis were performed using SAS 9.4 (SAS Institute, Gary, NC, USA) and R (R Development Core Team).

## Results

**Figure 1:**
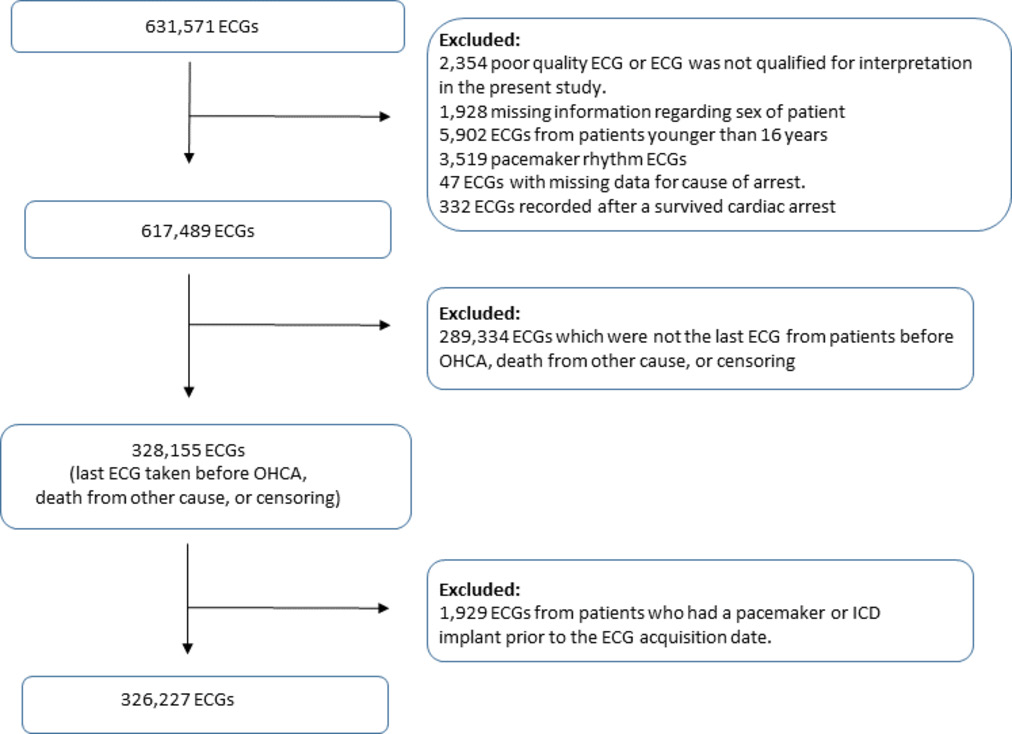
The selection process of the ECG study population. ICD, implantable cardioverter-defibrillator; OHCA, out-of-hospital cardiac arrest.

A total of 326 227 patients had an ECG recording during the study period and fulfilled the inclusion criteria (figure 1).

The total follow-up time of the study population was 1 506 274 person-years with a median follow-up time of 4.0 years (Q1–Q3: 2.2–6.6 years). During the follow-up period, 2667 patients suffered an OHCA, 52 735 died from other cause and 6136 emigrated before the end of the study period. The incidence of OHCA was 177 (95% CI 153 to 205) per 100 000 person-years.

Table 1 shows the clinical characteristics of the study population for patients suffering from OHCA, compared with patients who were censored or died. The median age of patients suffering from OHCA was 72.5 years (Q1–Q3: 62.3–80.7), compared with 78.8 (Q1–Q3: 69.2–85.4) years of patients who died from other cause and 53.1 years (Q1–Q3: 40.2–64.6) of censored patients. Patients suffering from OHCA were more frequently male than patients who were censored or died (61.9% vs 45.2% and 44.0%, respectively). Compared with patients who died, patients suffering from OHCA had more ECG abnormalities, except atrial fibrillation, atrial flutter, Cornell LVH, and RBBB. In general, patients suffering from OHCA had more cardiac comorbidities, except congenital heart disease, and more patients suffering from OHCA received pharmacological treatment than patients who died or were censored.

For the patients suffering from OHCA, 30.0% (801 of 2667) had a known cardiac disease at the time of the ECG recording, whereas 49.3% (1315 of 2667) of the patients had a known cardiac disease when the OHCA occurred. Notably, 14.2% of the patients without identified cardiac disease at the time of the OHCA had LBBB, NSIB or ST-depression without concomitant atrial fibrillation at the time of the ECG recording (table 2).

### Association between ECG abnormalities and OHCA

**Figure 2:**
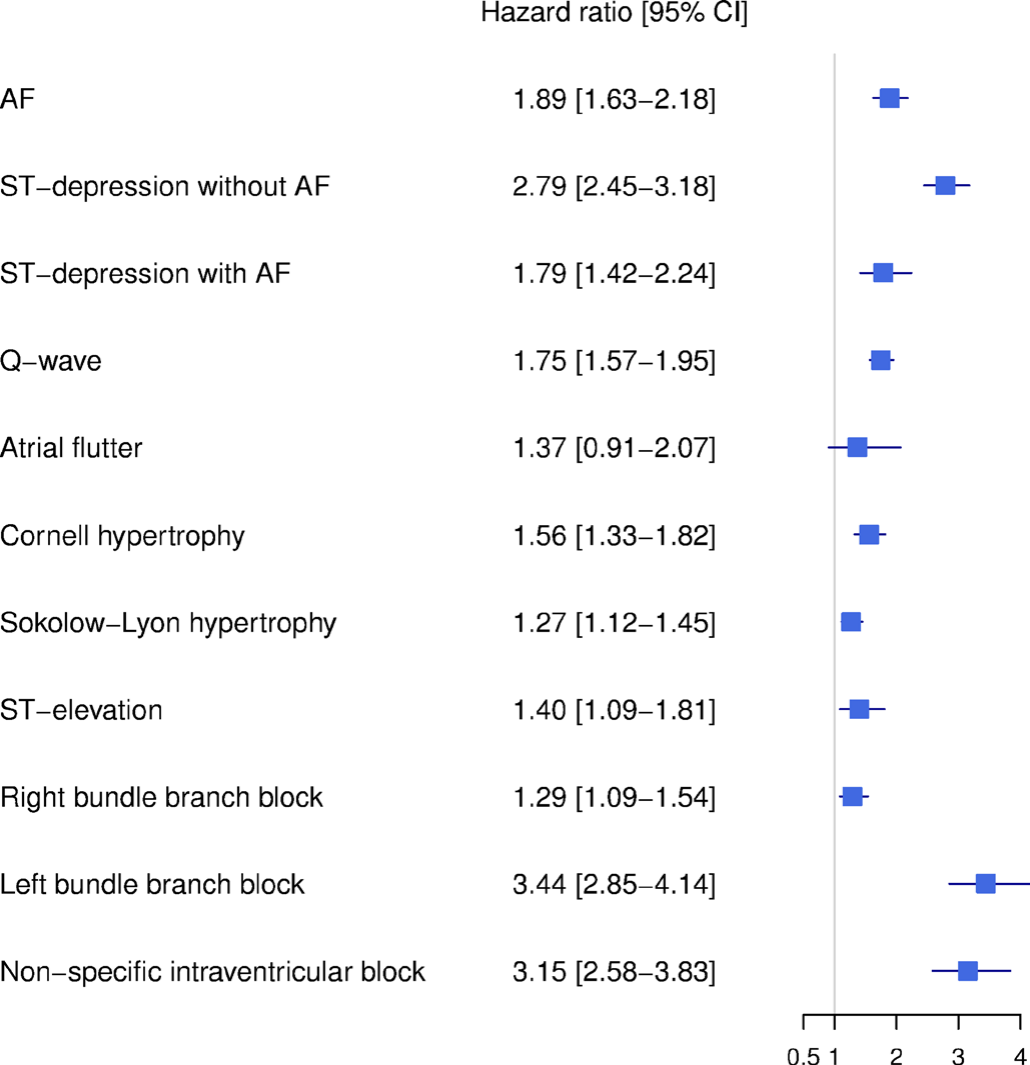
Multivariable Cox regression model showing the association between different ECG abnormalities and out-of-hospital cardiac arrest. Legend: The figure shows the results from the multivariable Cox regression model (AF, ST-depression, Q-wave, atrial flutter, Cornell hypertrophy, Sokolow-Lyon hypertrophy, ST-elevation, LBBB, RBBB and NSIB), adjusted for age and sex. An interaction was identified between AF and ST-depression (p<0.01 for interaction). AF, atrial fibrillation; LBBB, left bundle branch block; RBBB, right bundle branch block.

Figure 2 shows the results of the multivariable Cox model, adjusted for age and sex. Overall, the different ECG abnormalities were all associated with OHCA, except atrial flutter. The following ECG findings were strongly associated with OHCA: LBBB (HR 3.44; 95% CI 2.85 to 4.14) and NSIB (HR 3.15; 95% CI 2.58 to 3.83). Likewise, ST-depression without atrial fibrillation was highly associated with OHCA (HR 2.79; 95% CI 2.45 to 3.18), but the association between ST-depression and OHCA decreased when atrial fibrillation was concomitantly present in the ECG (HR 1.79; 95% CI 1.42 to 2.24, p<0.01 for interaction between ST-depression and atrial fibrillation). Male sex compared with female sex was associated with OHCA (HR 2.21; 95% CI 2.04 to 2.41).

Online supplementary material, figure 1 shows the results of the multivariable fully adjusted Cox model. Overall, the associations between the different ECG abnormalities and OHCA remained.

### Ten-year risk of OHCA

**Figure 3:**
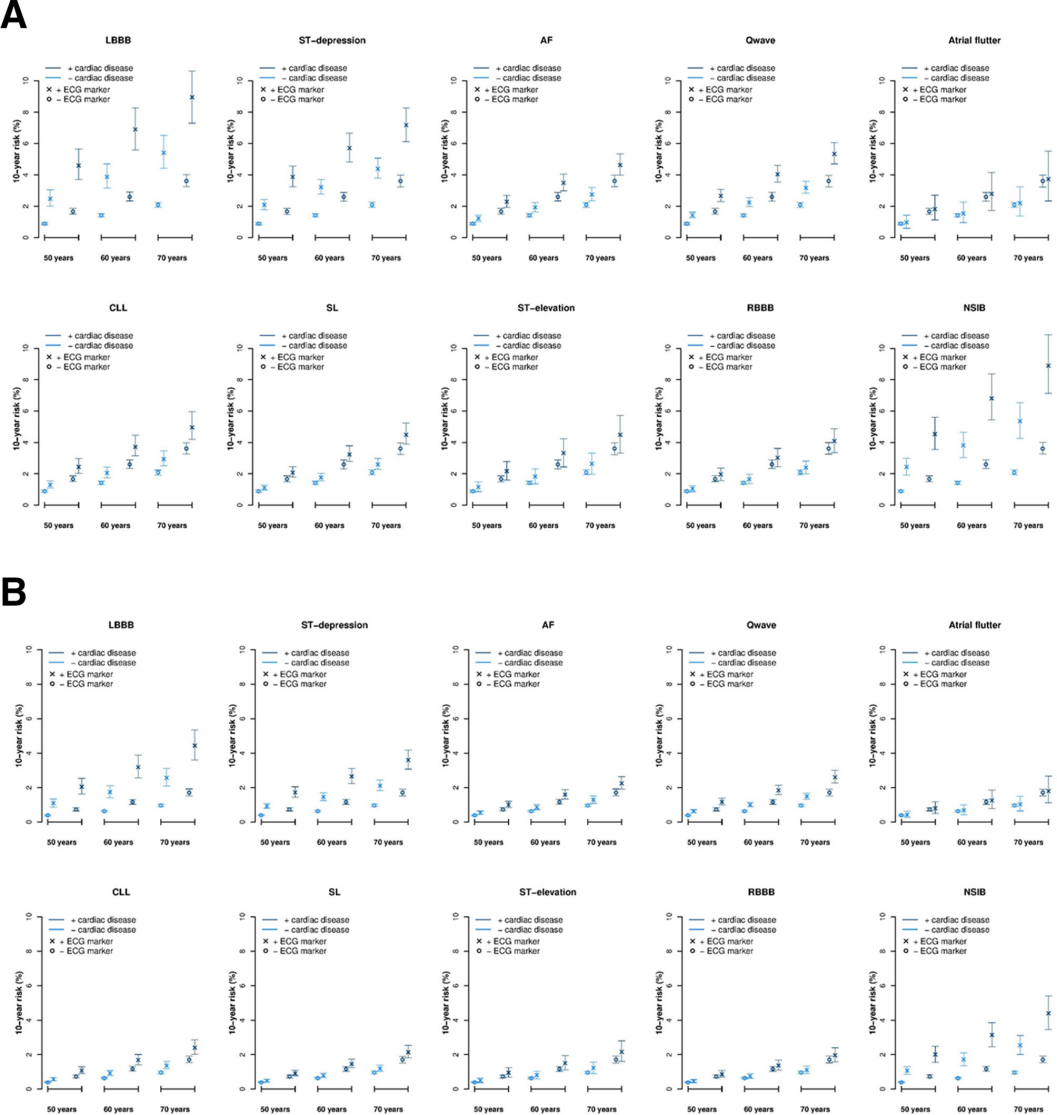
A+B Ten-year risks of OHCA according to sex, cardiac disease status, age, and the different ECG abnormalities. A+B legend: The figure shows the 10-year risks of suffering an OHCA for the different ECG abnormalities according to sex, whether or not the patient had known cardiac disease at the time of the ECG recording, and age at 50, 60 and 70 years. Cardiac disease included heart failure, ischaemic heart disease, prior myocardial infarction, cardiomyopathy, AF, atrial flutter, congenital heart disease, valvular heart disease, other cardiac arrhythmia, other cardiac disease. The analyses considered the competing risk of death from other cause. AF, atrial fibrillation; CLL, Cornell criteria of left ventricular hypertrophy; LBBB, left bundle branch block; NSIB, non-specific intraventricular; OHCA, out-of-hospital cardiac arrest.

Figure 3A, B shows the 10-year risks of OHCA for men and women with and without the different ECG abnormalities, known cardiac disease at ages 50, 60 and 70 years. For women, the 10-year risks of OHCA were overall half of the risks for males with the same ECG abnormalities (figure 3B). For both women and men, especially LBBB, ST-depression or NSIB posed a higher risk of OHCA compared with not having the ECG abnormalities. This was consistent for both patients with and without known cardiac disease. Online supplementary figure 2ab shows 5-year risk.

## Discussion

This study shows that several frequently encountered ECG abnormalities in a primary care setting were associated with increased risk of OHCA. Especially LBBB, NSIB and ST-depression without concomitant atrial fibrillation were strongly associated with OHCA. The 10-year absolute risk of OHCA was highest for patients having LBBB, NSIB and ST-depression with known cardiac disease. Furthermore, patients without cardiac disease but with LBBB, NSIB and ST-depression also had high absolute risks compared with patients not having these ECG abnormalities.

Previous studies have assessed the association between different ECG abnormalities and sudden death. Intraventricular block, including LBBB, has been described as a risk factor for sudden cardiac death and increased mortality.^6 23 24^ In our study, LBBB and NSIB were both highly associated with OHCA. Furthermore, some of the patients without known cardiac disease at the time of the OHCA event actually had these ECG abnormalities when the ECG was recorded. ST-depression has been associated with cardiovascular death and is a known indicator of myocardial ischaemia.^12 15^ In our study, ST-depression was strongly associated with OHCA but diminished when concomitantly present with atrial fibrillation. ST-depression during atrial fibrillation has been described as a poor predictor of coronary artery disease.^25^ Nonetheless, a recent study described an association between atrial fibrillation and non-ST-elevation myocardial infarction.^26^ Pathological Q-waves and Cornell or Sokolow-Lyon voltage criteria for LVH were not as strongly associated with OHCA as ST-depressions, LBBB and NSIB. Both are known to have poor sensitivity.^27 28^ This could explain our findings as both pathological Q-waves and LVH are known to be associated with increased risk of sudden death.^1 3 15^ Consequently, some patients with these ECG abnormalities may carry a high risk of OHCA, but the detection value is blurred by other low-risk patients having these ECG criteria without cardiac disease.

A recent study by Waks *et al* suggested that patients with a 10-year risk of sudden cardiac death between 1% and 5% should be considered at intermediate risk while patients with a 10-year risk >5% should be considered at high risk.^29^ In our study, 60-year and 70-year-old men with known cardiac disease and LBBB, ST-depression or NSIB had the highest risk of OHCA (10-year risk >5%). In addition, the risk of OHCA for 70-year-old men without known cardiac disease exceeded 5% when LBBB or NSIB was present in the ECG. Furthermore, 50-year-old men without known cardiac disease had an intermediate risk above 2% when one of the three ECG abnormalities was present in the ECG.

It has been reported that 40%–60% of all sudden cardiac deaths occur as the first manifestation of previously undetected heart disease.^2 3^ This was also the case in our study. This seriously challenges the option of taking preventive steps for reducing the occurrence of OHCA and sudden cardiac death. Screening for sudden cardiac death in general communities is not recommended in previous reports.^3 11^ Meanwhile, a recent study has shown that ECG abnormalities in asymptomatic middle-aged people predict fatal cardiac events.^30^ New risk models for predicting sudden cardiac death using advanced ECG parameters have been suggested, but clinical implementation has remained scarce.^29^ This could be due to the difficulty of implementation and interpretation of advanced ECG parameter in the primary care setting. This poses a problem for detecting patients at risk of sudden cardiac death, but without identified cardiac disease as such patients are rarely seen in secondary care settings. Even with no screening in the primary care setting, many patients have routine ECG examinations and knowledge about risk of sudden cardiac death when ECG abnormalities are encountered is essential for optimal handling of these patients. This is illustrated by the fact that 14% of the patients suffering from OHCA without known cardiac disease at the time of the OHCA had high-risk ECG abnormalities at baseline. For patients presenting with serious ECG abnormalities in a primary care setting, consistent with cardiac disease, preventive measures such as referral to a secondary care evaluation should be considered.

### Limitations

A major limitation of our study is the observational design. As such, our results should be interpreted only as associative and not causal relations to OHCA. In addition, the design precludes assurance that unmeasured potential confounders could have been present and biassed the results. Our study population is not necessarily generalisable to the general population. Our study population consisted of patients getting an ECG examination in a centralised primary care setting facility. The lack of generalisability is reflected in the higher incidence of OHCA, cardiovascular disease and all-cause mortality compared with the general population.^12 13 31^ However, this is not only a limitation as normal healthy people do not routinely undergo ECG recording and thus our study population is more likely to be representative of a real-life situation. Patients suffering from OHCA only included patients where a resuscitative attempt was performed excluding patients with late signs of death. Consequently, our incidence and risk estimations of OHCA are likely underestimations. Another major limitation of our study is that patient symptoms and the reasons why the ECG was requisitioned are unknown.

## Conclusions

This study shows that several common ECG findings in a primary care setting are associated with OHCA, especially LBBB, ST-depression without concomitant atrial fibrillation and NSIB. Furthermore, 14% of patients without known heart disease had these ECG abnormalities prior to the OHCA event.

## References

[1] Zipes DP, Wellens HJ, death Scardiac Sudden cardiac death.. Circulation 1998;98:2334–51. 10.1161/01.CIR.98.21.2334 9826323

[2] Hollenberg J, Svensson L, Rosenqvist M Out-of-hospital cardiac arrest: 10 years of progress in research and treatment. J Intern Med 2013;273:572–83. 10.1111/joim.12064 23480824

[3] Priori SG, Blomström-Lundqvist C, Mazzanti A, et al 2015 ESC guidelines for the management of patients with ventricular arrhythmias and the prevention of sudden cardiac death: the task Force for the management of patients with ventricular arrhythmias and the prevention of sudden cardiac death of the European Society of cardiology (ESC). endorsed by: association for European paediatric and congenital cardiology (AEPC). Eur Heart J 2015;36:2793–867. 10.1093/eurheartj/ehv316 26320108

[4] Rabkin SW, Mathewson FL, Tate RB The electrocardiogram in apparently healthy men and the risk of sudden death. Br Heart J 1982;47:546–52. 10.1136/hrt.47.6.546 6177327 PMC481180

[5] Okin PM, Devereux RB, Jern S, et al Regression of electrocardiographic left ventricular hypertrophy during antihypertensive treatment and the prediction of major cardiovascular events. JAMA 2004;292:2343–9. 10.1001/jama.292.19.2343 15547161

[6] Ashley EA, Raxwal V, Froelicher V An evidence-based review of the resting electrocardiogram as a screening technique for heart disease. Prog Cardiovasc Dis 2001;44:55–67. 10.1053/pcad.2001.24683 11533927

[7] Cullen K, Wearne KL, Stenhouse NS, et al Q waves and ventricular extrasystoles in resting electrocardiograms. A 16 year follow up in Busselton study. Br Heart J 1983;50:465–8. 10.1136/hrt.50.5.465 6196042 PMC481440

[8] Rabkin SW, Mathewson FA, Tate RB Natural history of left bundle-branch block. Br Heart J 1980;43:164–9. 10.1136/hrt.43.2.164 6444828 PMC482257

[9] Rose G, Baxter PJ, Reid DD, et al Prevalence and prognosis of electrocardiographic findings in middle-aged men. Br Heart J 1978;40:636–43. 10.1136/hrt.40.6.636 656238 PMC483461

[10] Auer R, Bauer DC, Marques-Vidal P, et al Association of major and minor ECG abnormalities with coronary heart disease events. JAMA 2012;307:1497–505. 10.1001/jama.2012.434 22496264 PMC4006989

[11] Chou R, Arora B, Dana T, et al Screening asymptomatic adults with resting or exercise electrocardiography: a review of the evidence for the U.S. Preventive Services Task Force. Ann Intern Med 2011;155:375–85. 10.7326/0003-4819-155-6-201109200-00006 21930855

[12] Rasmussen PV, Nielsen JB, Pietersen A, et al Electrocardiographic precordial ST-segment deviations and the risk of cardiovascular death: results from the Copenhagen ECG study. J Am Heart Assoc 2014;3:e000549 10.1161/JAHA.113.000549 24815495 PMC4309043

[13] Wissenberg M, Lippert FK, Folke F, et al Association of national initiatives to improve cardiac arrest management with rates of bystander intervention and patient survival after out-of-hospital cardiac arrest. JAMA 2013;310:1377–84. 10.1001/jama.2013.278483 24084923

[14] Healthcare G MarquetteTM 12SLTM ECG Analysis Program - Statement of Validation and Accuracy, 2008.

[15] Thygesen K, Alpert JS, Jaffe AS, et al Third universal definition of myocardial infarction. Circulation 2012;126:2020–35. 10.1161/CIR.0b013e31826e1058 22923432

[16] Deshpande A, Birnbaum Y ST-segment elevation: distinguishing ST elevation myocardial infarction from ST elevation secondary to nonischemic etiologies. World J Cardiol 2014;6:1067–79. 10.4330/wjc.v6.i10.1067 25349651 PMC4209433

[17] Wang K, Asinger RW, Marriott HJL ST-segment elevation in conditions other than acute myocardial infarction. N Engl J Med 2003;349:2128–35. 10.1056/NEJMra022580 14645641

[18] Sokolow M, Lyon TP The ventricular complex in right ventricular hypertrophy as obtained by unipolar precordial and limb leads. Am Heart J 1949;38:273–94. 10.1016/0002-8703(49)91335-6 18133359

[19] Casale PN, Devereux RB, Alonso DR, et al Improved sex-specific criteria of left ventricular hypertrophy for clinical and computer interpretation of electrocardiograms: validation with autopsy findings. Circulation 1987;75:565–72. 10.1161/01.CIR.75.3.565 2949887

[20] Hancock EW, Deal BJ, Mirvis DM, et al AHA/ACCF/HRS recommendations for the standardization and interpretation of the electrocardiogram: Part V: electrocardiogram changes associated with cardiac chamber hypertrophy: a scientific statement from the American Heart Association electrocardiography and arrhythmias Committee, Council on clinical cardiology; the American College of cardiology Foundation; and the heart rhythm society: endorsed by the International Society for computerized Electrocardiology. Circulation 2009;119:e251–61. 10.1161/CIRCULATIONAHA.108.191097 19228820

[21] Schmidt M, Schmidt SAJ, Sandegaard JL, et al The Danish national patient Registry: a review of content, data quality, and research potential. Clin Epidemiol 2015;7:449–90. 10.2147/CLEP.S91125 26604824 PMC4655913

[22] Benichou J, Gail MH Estimates of absolute cause-specific risk in cohort studies. Biometrics 1990;46:813–26. 10.2307/2532098 2242416

[23] Nakamura Y, Okamura T, Inohara T, et al Prognostic values of bundle branch blocks for cardiovascular mortality in Japanese (24year follow-up of NIPPON DATA80). J Electrocardiol 2013;46:360–5. 10.1016/j.jelectrocard.2013.03.009 23597404

[24] Aro AL, Anttonen O, Tikkanen JT, et al Intraventricular conduction delay in a standard 12-lead electrocardiogram as a predictor of mortality in the general population. Circ Arrhythm Electrophysiol 2011;4:704–10. 10.1161/CIRCEP.111.963561 21841194

[25] Pradhan R, Chaudhary A, Donato AA Predictive accuracy of ST depression during rapid atrial fibrillation on the presence of obstructive coronary artery disease. Am J Emerg Med 2012;30:1042–7. 10.1016/j.ajem.2011.06.027 21855255

[26] Soliman EZ, Lopez F, O'Neal WT, et al Atrial fibrillation and risk of ST-segment-elevation versus Non-ST-Segment-Elevation myocardial infarction: the Atherosclerosis Risk in Communities (ARIC) study. Circulation 2015;131:1843–50. 10.1161/CIRCULATIONAHA.114.014145 25918127 PMC4447576

[27] Okin PM, Roman MJ, Devereux RB, et al Time-voltage QRS area of the 12-lead electrocardiogram: detection of left ventricular hypertrophy. Hypertension 1998;31:937–42. 9535418 10.1161/01.hyp.31.4.937

[28] Nadour W, Doyle M, Williams RB, et al Does the presence of Q waves on the EKG accurately predict prior myocardial infarction when compared to cardiac magnetic resonance using late gadolinium enhancement? A cross-population study of noninfarct vs infarct patients. Heart Rhythm 2014;11:2018–26. 10.1016/j.hrthm.2014.07.025 25063692

[29] Waks JW, Sitlani CM, Soliman EZ, et al Global electric heterogeneity risk score for prediction of sudden cardiac death in the general population: the Atherosclerosis Risk in Communities (ARIC) and cardiovascular health (CHS) studies. Circulation 2016;133:2222–34. 10.1161/CIRCULATIONAHA.116.021306 27081116 PMC4899170

[30] Terho HK, Tikkanen JT, Kenttä TV, et al The ability of an electrocardiogram to predict fatal and non-fatal cardiac events in asymptomatic middle-aged subjects. Ann Med 2016;48:525–31. 10.1080/07853890.2016.1202442 27684209

[31] Nielsen JB, Graff C, Pietersen A, et al J-shaped association between QTc interval duration and the risk of atrial fibrillation: results from the Copenhagen ECG study. J Am Coll Cardiol 2013;61:2557–64. 10.1016/j.jacc.2013.03.032 23583581

