# Supplementary material for: Associations between common ECG abnormalities and out-of-hospital cardiac arrest
Source: Open Heart. 2019 May 21;6(1):e000905. doi: 10.1136/openhrt-2018-000905 (PMC6546195; doi:10.1136/openhrt-2018-000905)
Supplement: Supplementary data [file openhrt-2018-000905supp001.pdf]

Supplementary material

Page 2 – Additional description of ECG variables analyzed

Page 3-8 – Detailed list of ICD10 and ATC codes used to define comorbidities and medicine use.

Page 9-12 – Additional analysis

Page 13 – Accuracy of the 12SL Marquette algorithm

Page 14 – References

Additional description of ECG variables analyzed

The definition of ST-elevation was slightly modified as the measurement of the ST segment was performed at QRS offset plus 1/16 of the average RR interval known as the STM point measure in the 12SL algorithm (equivalent to about 80 ms after QRS offset in most cases). This measurement point was selected instead of the J-point because a notched or slurred appearance of the terminal QRS complex (also described as early repolarization) can make it difficult to define the J-point.[1] LBBB and NSIB are known to affect the repolarization of the heart causing ST-deviations.[2–4] Consequently, when ST-T deviation was concomitantly present with LBBB or NSIB we disregarded the finding. In patients with RBBB, ST-T deviations in V1-V3 are common.[4] When ST-T deviations in V1-V3 were present together with RBBB the ST-T deviations were disregarded.

Sokolow-Lyon and Cornell sex-specific voltage criteria were used to identify ECG LVH .[5,6] The criteria for LVH have low predictive value when applied on an ECG with identified LBBB, RBBB and NSIB.[7] ST-T deviations together with ECGs with hypertrophy have been associated with larger left ventricular mass and risk of cardiovascular disease.[7] Consequently, if LBBB, RBBB, or NSIB were identified, ECGs were not assigned the Sokolow-Lyon and Cornell LVH criteria.

ST-T deviations together with the hypertrophy criteria were acknowledged and included in the analysis. In accordance with the Third Universal Myocardial Infarction Definition, pathologic Q-waves, as a sign of prior myocardial infarction, were only defined when hypertrophy criteria or LBBB were absent.[4]

Detailed list of ICD-10 and ATC codes used for defining comorbidity and medication

Table S1: ICD-10 codes of comorbidities

Disease	ICD10	Years before baseline ECG
**Cardiac comorbidities**		
Ischemic heart disease	I20, I25	5
Previous myocardial infarction	I21, I22, I23, I24	5
Cardiomyopathy	I119, I517, I42, I43	5
Heart failure	I110, I50	5
Valvular heart disease	I34, I35, I36, I37	5
Congenital heart disease	Q20, Q21, Q22, Q23, Q24, Q25	5
Atrial fibrillation/atrial flutter	I48	5
Other cardiac arrhythmias	I44, I45, I47, I49	5
Other cardiac disease	I30, I31, I32, I33, I38, I39, I40, I41, I51, I52	5
**Non-cardiac comorbidities**		
Cerebrovascular disease	I60, I61, I62, I63, I64, I65, I66, I67, I68, I69	5
Perifer vascular disease	I70, I71, I72, I74, R02	5
Malignant disease	C00-C97	5

Renal disease	N03, N04, N17, N18, N19, R34, I12, I13, Z992	5
Liver disease	K70, K71, K72, K73, K74, K75, K76, K77	5
Chronic obstructive pulmonary disease	J41, J42, J43, J44	5

Table S2: ATC codes of medication

**Medications**	**ATC code**	**Days before OHCA**
QT prolonging medication*	See Table 3	180
Glucose lowering medicine	C01D	180
Beta blockers	C01B	180
Diuretics	C07	180
Angiotensin converting enzyme inhibitors and angiotensin receptor blockers	C08	180
Calcium channel blockers	C01AA05	180

* QT interval prolonging drugs were acquired from www.qtdrugs.org (accessed February 26, 2016).

**Table S3: ATC codes of QT prolonging medication (detailed list)***	
**QT prolonging medication (Main ATC groups)**	**ATC code**
Cardiovascular system	**Amiodarone C01BD01, disopyramide C01BA03, dofetilide C01BD04, Dronedarone C01BD07, flecainide C01BC04, ibutilide C01BD05, procainamide C01BA02, quinidine C01BA01, Sotalol C07AA07,**<br>**Probucol C10AX02, Bepridil C08EA02**<br>Ranolazine C01EB18, Isradipine C08CA03, Moexipril C09AA13, Nicardipine C08CA04
Genito-urinary system and sex hormones	**Papaverine G04BE02,** Alfuzosin G04CA01, Mirabegron G04BD12, Tolterodine G04BD07, Vardenafil G04BE09, Apomorphine G04BE07, Mifepristone G03XB01
Antineoplastic and immunomodulating agents	**Arsenic trioxide L01XX27, Vandetanib L01XE12, Anagrelide L01XX35, Oxaliplatin L01XA03,**<br>Dabrafenib L01XE23, Eribulin L01XX41, Lapatinib L01XE07, Nilotinib L01XE08, Sunitinib L01XE04, Tamoxifen L02BA01, Vorinostat L01XX38, Toremifene L02BA02, Crizotinib L01XE16, Vemurafenib L01XE15, Bortezomib L01XX32, Bosutinib L01XE14, Dasatinib L01XE06, Pazopanib L01XE11, Sorafenib L01XE05, Ceritinib L01XE28, Degarelix L02BX02, Leuprorelin L02AE02,

	Lenvatinib L01XE29, Tacrolimus L04AD02,<br>Panobinostat L01XX42, Fingolimod L04AA27
Nervous system	**Propofol N01AX10,**<br>**Cocaine N01BC01, sevoflurane N01AB08,**<br>**Methadone N07BC02, levomethadone N07BC05,**<br>**Citalopram N06AB04, Escitalopram N06AB10,**<br>**Haloperidol N05AD01, Mesoridazine N05AC03,**<br>**Pimozide N05AG02, Thioridazine N05AC02,**<br>**Chlorpromazine N05AA01, levomepromazine**<br>**N05AA02, droperidol N05AD08, Sulpiride**<br>**N05AL01, Donepezil N06DA02,** Atmoxetine<br>N06BA09, Felbamate N03AX10, Apomorphine<br>N04BC07, Tetrabenazine N07XX06,<br>Dexmedetomidine N05CM18,<br>Venlafaxine N06AX16, Asenapine N05AH05,<br>Iloperidone N05AX14, Aripiprazole N05AX12,<br>Clozapine N05AH02, Olanzapine N05AH03,<br>Paliperidone N05AX13, Risperidone N05AX08,<br>Sertindole N05AE03, Pipamperone N05AD05,<br>Lithium N05AN01, Clomipramine N06AA04,<br>Desipramine N06AA01, Imipramine N06AA02,<br>Mirtazapine N06AX11, Nortriptyline N06AA10,<br>Trimipramine N06AA06, Cyamemazine N05AA06

Alimentary tract and metabolism	**Ondansetron A04AA01, Domperidone A03FA03, Cisapride A03FA02, Papaverine A03AD01,** Dolasetron A04AA04, Tropisetron A04AA03, Granisetron A04AA02, Famotidine A02BA03
Respiratory system	**Astemizole R06AX11, Terfenadine R06AX12,** Promethazine R06AD02, Hydrocodone R05DA03
Antiparasitic products, insecticides and repellents	**Chloroquine P01BA01, Halofantrine P01BX01, Pentamidine P01CX01,** Artenimol and piperaquine P01BF05
Blood and blood forming organs	**Cilostazol B01AC23,**
Antiinfectives for systemic use	**Azithromycin J01FA10, Clarithromycin J01FA09, Erythromycin J01FA01, grepafloxacin J01MA11, levofloxacin J01MA12, Moxifloxacin J01MA14, Gatifloxacin J01MA16, Sparfloxacin J01MA09, Ciprofloxacin J01MA02, Fluconazole J02AC01,** Atazanavir J05AE08, Foscarnet J05AD01, Rilpivirine J05AG05, Saquinavir J05AE01, Bedaquiline J04AK05, Delamanid J04AK06, Gemifloxacin J01MA15, Norfloxacin J01MA06, Ofloxacin J01MA01, Roxithromycin J01FA06, Telavancin J01XA03, Telithromycin J01FA15
Systemic hormonal preparations, excluding sex hormones and insulins	Oxytocin H01BB02, Pasireotide H01CB05
Musculo-skeletal system	Tizanidine M03BX02

Various	Perflutren lipid microspheres V08DA06

* The list of QT interval prolonging drugs were defined according to the list of QT prolonging drugs at www.qtdrugs.org (accessed February 26, 2016).

Drugs highlighted with **bold** text are drugs classified with known risk of torsades de pointe, while the other drugs are classified with a possible risk of torsades de pointe.

Additional analysis

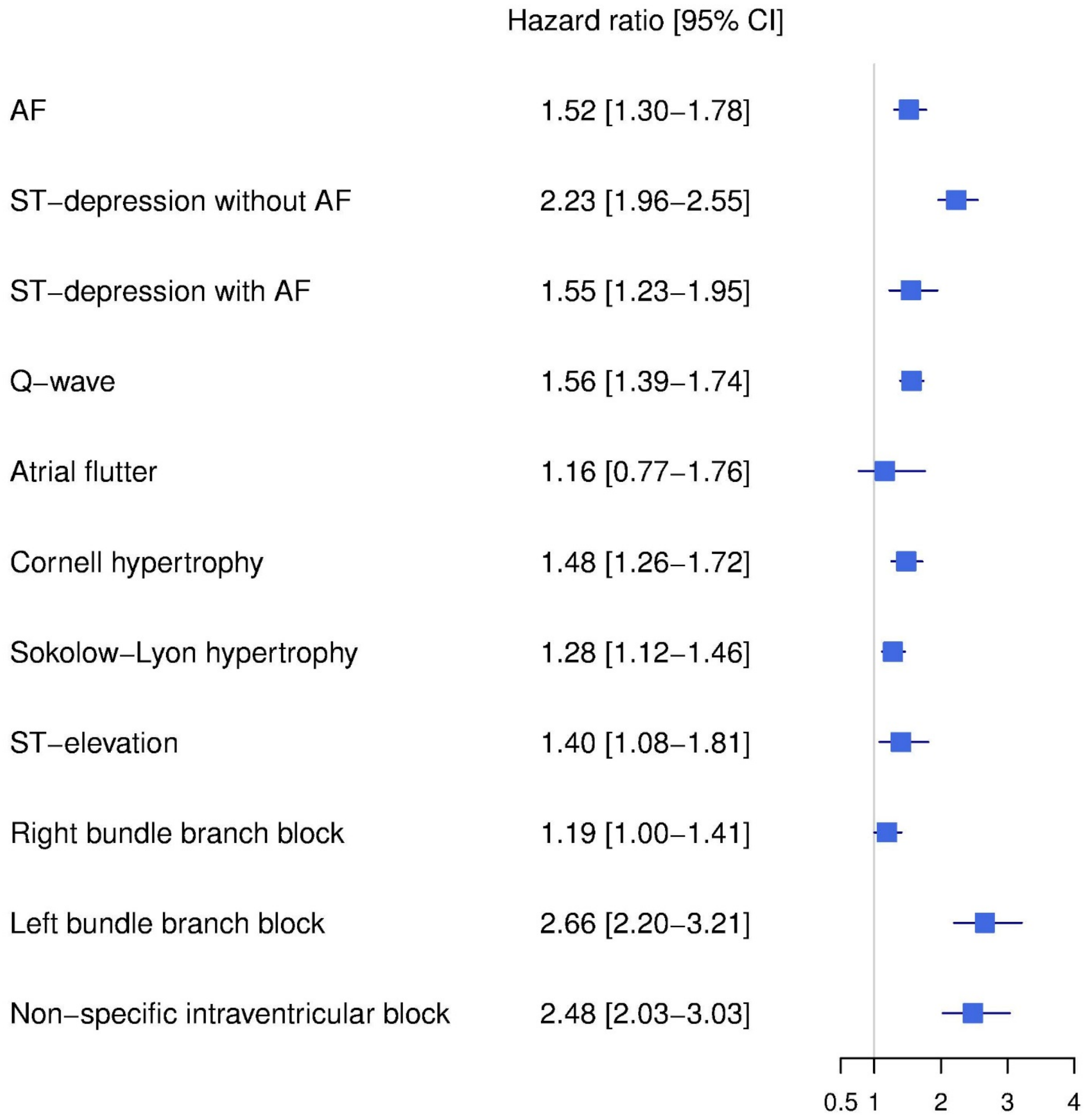

Figure 1 title: Fully adjusted multivariable cox regression model showing the association between different ECG abnormalities and out-of-hospital cardiac arrest.

Figure 1 legend: The figure shows the results from the multivariable cox regression model (atrial fibrillation, ST-depression, Q-wave, atrial flutter, Cornell hypertrophy, Sokolow-Lyon hypertrophy, ST-elevation, LBBB, RBBB and non-specific intraventricular block). The model was adjusted for age, sex, heart failure, ischemic

heart disease, prior myocardial infarction, cardiomyopathy, atrial fibrillation, atrial flutter, congenital heart disease, valvular heart disease, other cardiac arrhythmia, other cardiac disease, cerebro vascular disease, perifer vascular disease, chronic obstructive pulmonary disease, malignant disease, renal disease, liver disease, QTc prolonging medication, glucose lowering medication, beta-blockers, diuretics, ACE inhibitor/angiotensin II receptor blockers, calcium inhibitors.

HR hazard ratio; 95% CI – 95% confidence interval; AF atrial fibrillation; LBBB left bundle branch block; RBBB right bundle branch block.

Five-year risk of out-of-hospital cardiac arrest

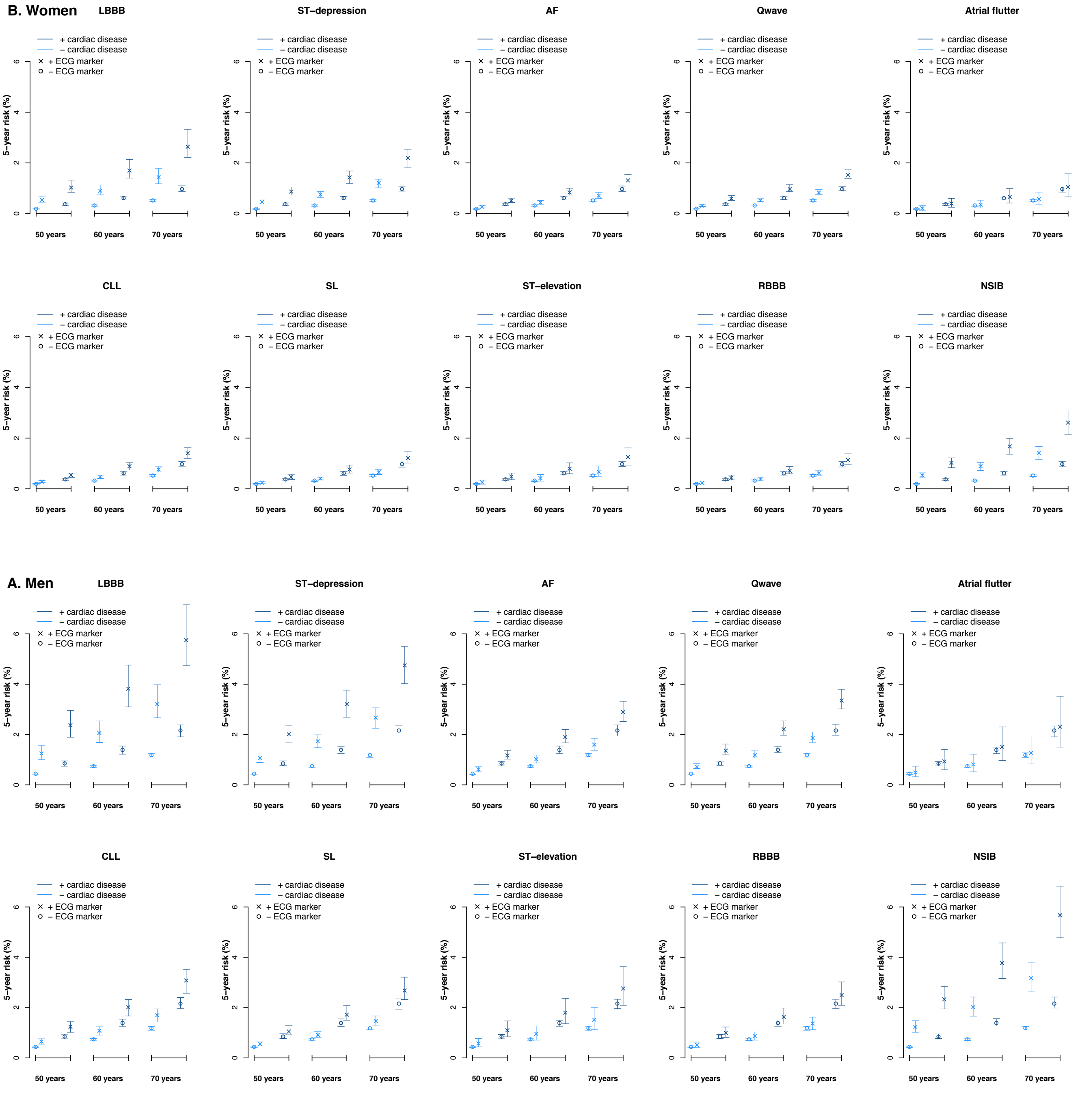

Figure 2

Figure 2A+B title: Ten-year risks of out-of-hospital cardiac arrest according to sex, cardiac disease status, age, and the different ECG abnormalities

Figure 2A+B legend: The figure shows the 5-year risks of suffering an out-of-hospital cardiac arrest for the different ECG abnormalities according to sex, whether or not the patient had known cardiac disease at the time of the ECG recording, and age at 50, 60 and 70 years. Cardiac disease included: Heart failure, ischemic heart disease, prior myocardial infarction, cardiomyopathy, atrial fibrillation, atrial flutter, congenital heart disease, valvular heart disease, other cardiac arrhythmia, other cardiac disease. The analyses considered the competing risk of death from other cause.

AF atrial fibrillation; CLL Cornell criteria of left ventricular hypertrophy; LBBB left bundle branch block; NSIB non-specific intraventricular

Accuracy of the 12SL Marquette algorithm

The accuracy of the 12SL Marquette algorithm is described thoroughly.[8] Atrial fibrillation has been found to have a sensitivity ranging from 76.1%-90.8% and a specificity of 98.9-99.6%. Atrial flutter has a sensitivity ranging from 61.0%-65.9% and a specificity of 99.9%, Left Bundle Branch Block a sensitivity from 78%-90.9% and a specificity from 99.9%-100%. Right Bundle Branch Block has been found to have a sensitivity ranging from 90%-93.2% and a specificity of 99.8%-100%.[8]

Data on the accuracy of 12SL measurements we refer to the GE Marquette 12SL ECG Analysis Program Physician's Guide.[8]

Supplementary material references

1 Rasmussen PV, Nielsen JB, Pietersen A, *et al.* Electrocardiographic precordial ST-segment deviations and the risk of cardiovascular death: results from the Copenhagen ECG Study. *J Am Heart Assoc* 2014;**3**:e000549. doi:10.1161/JAHA.113.000549

2 Deshpande A, Birnbaum Y. ST-segment elevation: Distinguishing ST elevation myocardial infarction from ST elevation secondary to nonischemic etiologies. *World J Cardiol* 2014;**6**:1067–79. doi:10.4330/wjc.v6.i10.1067

3 Wang K, Asinger RW, Marriott HJL. ST-segment elevation in conditions other than acute myocardial infarction. *N Engl J Med* 2003;**349**:2128–35. doi:10.1056/NEJMra022580

4 Thygesen K, Alpert JS, Jaffe AS, *et al.* Third universal definition of myocardial infarction. *Circulation* 2012;**126**:2020–35. doi:10.1161/CIR.0b013e31826e1058

5 Sokolow M, Lyon TP. The ventricular complex in right ventricular hypertrophy as obtained by unipolar precordial and limb leads. *Am Heart J* 1949;**38**:273–94.

6 Casale PN, Devereux RB, Alonso DR, *et al.* Improved sex-specific criteria of left ventricular hypertrophy for clinical and computer interpretation of electrocardiograms: validation with autopsy findings. *Circulation* 1987;**75**:565–72.

7 Hancock EW, Deal BJ, Mirvis DM, *et al.* AHA/ACCF/HRS recommendations for the standardization and interpretation of the electrocardiogram: part V: electrocardiogram changes associated with cardiac chamber hypertrophy: a scientific statement from the American Heart Association Electrocardiography. *Circulation* 2009;**119**:e251-61. doi:10.1161/CIRCULATIONAHA.108.191097

8 Healthcare G. Marquette™ 12SL™ ECG Analysis Program - Statement of Validation and Accuracy. 2008.
